# Krüppel-Like Factor 6 Silencing Prevents Oxidative Stress and Neurological Dysfunction Following Intracerebral Hemorrhage *via* Sirtuin 5/Nrf2/HO-1 Axis

**DOI:** 10.3389/fnagi.2021.646729

**Published:** 2021-06-03

**Authors:** Jia Sun, Jinzhong Cai, Junhui Chen, Siqiaozhi Li, Xin Liao, Yixuan He, Xudong Chen, Sean Hu

**Affiliations:** ^1^Shenzhen Beike Biotechnology Research Institute, Shenzhen, China; ^2^Intervention and Cell Therapy Center, Shenzhen Hospital of Peking University, Shenzhen, China; ^3^Department of Interventional Radiology, Second Clinical Medical College of Jinan University (Shenzhen People's Hospital), Shenzhen, China

**Keywords:** intracerebral hemorrhage, nuclear transcription factor, Krüppel like factor 6, sirtuin 5, Nrf2, HO-1, nerve injury, oxidative stress

## Abstract

As a severe neurological deficit, intracerebral hemorrhage (ICH) is associated with overwhelming mortality. Subsequent oxidative stress and neurological dysfunction are likely to cause secondary brain injury. Therefore, this study sought to define the role of Krüppel-like factor 6 (KLF6) and underlying mechanism in oxidative stress and neurological dysfunction following ICH. An *in vivo* model of ICH was established in rats by injection of autologous blood, and an *in vitro* ICH cell model was developed in hippocampal neurons by oxyhemoglobin (OxyHb) exposure. Next, gain- and loss-of-function assays were performed *in vivo* and *in vitro* to clarify the effect of KLF6 on neurological dysfunction and oxidative stress in ICH rats and neuronal apoptosis and mitochondrial reactive oxygen species in OxyHb-induced hippocampal neurons. KLF6, nuclear factor erythroid 2–related factor 2 (Nrf2), and heme oxygenase 1 (HO-1) were highly expressed in hippocampal tissues of ICH rats, whereas sirtuin 5 (SIRT5) presented a poor expression. Mechanistically, KLF6 bound to the SIRT5 promoter and transcriptionally repressed SIRT5 to activate the Nrf2/HO-1 signaling pathway. KLF6 silencing alleviated neurological dysfunction and oxidative stress in ICH rats and diminished oxidative stress and neuronal apoptosis in OxyHb-induced neurons, whereas SIRT5 overexpression negated its effect. To sum up, KLF6 silencing elevated SIRT5 expression to inactivate the Nrf2/HO-1 signaling pathway, thus attenuating oxidative stress and neurological dysfunction after ICH.

## Introduction

Intracerebral hemorrhage (ICH), although accounts for only 15% of all strokes, disproportionally results in 50% of stroke-related mortality and disability all over the world (Planton et al., 2017). Risk factors for ICH include hypertension, history of heart attack, lipid-lowering medication, and fatigue (Sallinen et al., 2020). Researches on therapeutic intervention of ICH generally focus on arresting hemorrhage expansion including rapid blood pressure reduction, platelet transfusion, and clot evacuation (Ziai and Carhuapoma, 2018). Recent years have witnessed an impressive growth in our understanding of the oxidative stress and inflammatory reaction underlying pathophysiological process of ICH (Duan et al., 2016). It has been identified that oxidative stress is responsible for the damage after ICH, and therapeutic strategies related to molecular targets in antioxidant therapy are expected (Duan et al., 2016). Hence, this study was designed to investigate the effect of nuclear transcription factor Krüppel-like factor 6 (KLF6) on neurological dysfunction and oxidative stress in ICH.

KLF6 as a zinc finger transcription factor belongs to KLF family and is involved in various processes, such as organismal development, cell differentiation, function, and apoptosis (Kopp, 2015). A prior study validated that KLF6 played a vital role in inflammatory and hypoxic response (Kim et al., 2020). Moreover, it was reported that KLF6 was a key transcriptional factor in hippocampal neurons and mediated neuronal cell apoptosis (Salma and McDermott, 2012). Besides, KLF6 was verified to bind to the promoter region of sirtuin 5 (SIRT5) as a transcriptional repressor during adipocytes differentiation (Hong et al., 2019). SIRT5, as one member of sirtuin family of nicotinamide adenine dinucleotide–dependent histone deacetylases located in the mitochondria, is known as a key mediator of metabolic process, cellular apoptosis, and reactive oxygen species (ROS) (Liu et al., 2013; Singh et al., 2018). Additionally, an emerging research identified the crucial role of SIRT5 in brain metabolism on the basis of its contribution to ischemic tolerance (Koronowski et al., 2018). In addition, it was certified that SIRT5 mediated cisplatin resistance in ovarian cancer by regulating the nuclear factor erythroid 2–related factor 2 (Nrf2)/heme oxygenase 1 (HO-1) pathway (Sun et al., 2019). Specifically, Nrf2 and HO-1 are both recognized as promoter of the antioxidant and anti-inflammatory response thereby exerting cytoprotective effects to maintain cellular redox homeostasis (Zolnourian et al., 2019; Xie et al., 2020). Further, Nrf2/HO-1 signaling pathway was also recognized as crucial players in ICH management (Wei et al., 2017). In this context, it can be speculated that the KLF6/SIRT5/Nrf2/HO-1 axis may be implicated in the oxidative stress and neurological dysfunction after ICH. Therefore, animal and cell experiments were implemented in our research to confirm this hypothesis, thus providing a novel insight into potential mechanism in ICH.

## Materials and Methods

### Ethics Statement

Animal experiments were approved by the Animal Care and Use Committee of Shenzhen Beike Biotechnology Research Institute and in strict accordance with the recommendations in the Guide for the Care and Use of Laboratory Animals of the National Institutes of Health. Efforts were made to avoid unnecessary distress to the animals.

### Bioinformatics Analysis

The mRNA expression dataset GSE149317 of cerebral hemorrhage tissues was obtained through Gene Expression Omnibus (GEO) database (https://www.ncbi.nlm.nih.gov/geo/), including eight normal control brain tissues and eight brain tissues from ICH patients. The differential analysis was conducted using the “edgeR” software package of R language, and differentially expressed genes (DEGs) were screened out with the threshold of |log fold change| > 0.3 and *p* < 0.05. The downstream genes of transcription factor KLF6 were predicted using the JASPAR (http://jaspar.genereg.net/) and CistromeDB databases (http://cistrome.org/).

### *In vivo* ICH Rat Model Establishment

Adult male Sprague–Dawley (SD) rats (Shandong Laboratory Animal Center, Jinan, Shandong, China; weighing 280–300 g) were kept at a constant ambient temperature (22 ± 1°C) with a 12-h light–dark cycle. After rats were subjected to inhalation anesthesia by 3% pentobarbital sodium, the femoral artery was separated, and 50 μL of blood was collected for subsequent use. Rats were fixed in a stereotaxic device (RWD Life Science, Shenzhen, Guangdong, China), and the right basal ganglia (0.2 mm after bregma, 3 mm on the right side, 5.8 mm in depth) of rats were injected with 50 μL autologous blood within 10 min using a microsyringe pump at a uniform rate (5 μL/min). The needle was slowly withdrawn after it was retained for 10 min to prevent blood reflux, and then the scalp was sutured. Rats recovered alone in the cage and were allowed to free access to food and water. During the operation, the body temperature of rats was maintained at 37.0 ± 0.5°C. Sham-operated rats went through the same surgical operation only without blood injection.

### Experimental Design

A total of 42 rats (42 of 48 rats survived the ICH surgery) were randomly and equally allocated into seven groups (*n* = 6 per group) (**Figure 2A**): the normal group (SD rats without any treatment), the sham group (rats subjected to sham-operation), and five groups of ICH at different time points after ICH surgery (6, 12, 24, 72 h, and 7 days).

Next, rats after ICH modeling were anesthetized with 3% pentobarbital sodium and fixed in a stereotaxic device (RWD Life Science), and 15 μL of 1 μg/μL lentivirus harboring short hairpin RNA (sh)-KLF6, overexpression (oe)-SIRT5, sh-SIRT5, and their negative controls (sh-NC, oe-NC) were injected into the lateral ventricle (1.0 mm after bregma, 2.0 mm on the right side, 3.5 mm in depth) at a uniform rate (5 μL/min) within 10 min; the needle was slowly withdrawn after it was retained for 10 min to prevent blood reflux, and then the scalp was sutured.

Rats were assigned into the following groups (*n* = 6) by injecting different lentiviruses (**Figure 8A**): control group (SD rats without any treatment), sham group (rats subjected to sham operation), ICH group (rats subjected to ICH modeling), ICH + sh-NC group (rats subjected to ICH modeling and injection of lentivirus-mediated sh-NC), ICH + sh-KLF6 group (rats subjected to ICH modeling and injection of lentivirus-mediated sh-KLF6), ICH + sh-SIRT5 group (rats subjected to ICH modeling and injection of lentivirus-mediated sh-SIRT5), ICH + oe-NC group (rats subjected to ICH modeling and injection of lentivirus-mediated oe-NC), ICH + oe-SIRT5 group (rats subjected to ICH modeling and injection of oe-SIRT5), and ICH + sh-KLF6 + sh-SIRT5 group (rats subjected to ICH modeling and injection of lentivirus-mediated sh-KLF6 and sh-SIRT5). At the scheduled time, the rats were euthanized, and their brain tissues from the ICH-injured hemisphere were collected for analysis.

### *In vitro* ICH Cell Model Establishment

Primary hippocampal neurons were isolated from the hippocampal tissues of fetal rats (aged 16–18 days). The meninges and blood vessels were removed, and then the hippocampal tissues were treated with 0.25% trypsin with ethylenediaminetetraacetic acid (EDTA) for 5 min at 37°C and then washed three times with phosphate-buffered solution (PBS) to terminate the trypsinization. Next, the hippocampal tissue suspension was centrifuged at 1,000 × *g* for 5 min, and the cells were resuspended in neural basal medium containing 2% B27, 2 mM l-glutamine, 50 U/mL penicillin, and 50 U/mL streptomycin (all from GIBCO BRL, Grand Island, NY, USA). Finally, cells were settled in a six-well plate with fresh medium, and half of the medium was replaced every 2 days. An *in vitro* ICH model was established by exposing neurons to oxyhemoglobin (OxyHb) (20 μL) at 37°C with 5% CO_2_ for 6 h for subsequent experiments.

### Cell Culture and Treatment

Human embryonic kidney cells 293T were obtained from American Type Culture Collection (ATCC, Manassas, VA, USA) and cultured in a Dulbecco modified Eagle medium (Gibco-BRL) supplemented with 10% (vol/vol) fetal bovine serum. Primary hippocampal neurons were cultured with a neural basal medium (Gibco-BRL) containing 2% B27, 2 mM l-glutamine, 50 U/mL penicillin, and 50 U/mL streptomycin in an incubator with 5% CO_2_ at 37°C.

A lentiviral packaging system was constructed through LV5-GFP (lentiviral gene overexpression vector) and pSIH vector (lentiviral gene silencing vector); 293T cells were cotransduced with the packaging virus and the target vector, and the supernatant was collected after 48 h of culture. The supernatant contained virus particles after filtration and centrifugation, and the virus titer was detected. Viruses in logarithmic growth phase were collected. sh-NC, sh-KLF6, sh-SIRT5, oe-NC, and oe-SIRT5 were all synthesized by GenePharma Biological Co., Ltd. (Shanghai, China). The hippocampal neurons were assigned into the following groups: control group (neurons without any treatment), OxyHb group (neurons exposed to 20 μM OxyHb for 24 h), OxyHb + sh-NC group (neurons exposed to 20 μM OxyHb for 24 h after 48-h infection with sh-NC), OxyHb + sh-KLF6 group (neurons exposed to 20 μM OxyHb for 24 h after 48-h infection with sh-KLF6), OxyHb + sh-SIRT5 group (neurons exposed to 20 μM OxyHb for 24 h after 48-h infection with sh-SIRT5), OxyHb + oe-NC group (neurons exposed to 20 μM OxyHb for 24 h after 48-h transduction with oe-NC), OxyHb + oe-SIRT5 group (neurons exposed to 20 μM OxyHb for 24 h after 48-h transduction with oe-SIRT5), and OxyHb + sh-KLF6 + sh-SIRT5 group (neurons exposed to 20 μM OxyHb for 24 h after 48-h infection with sh-KLF6 + sh-SIRT5). When hippocampal neurons reached the logarithmic growth phase, they were trypsinized and titrated to prepare 5 cells/mL cell suspension, which was seeded in a six-well plate at a density of 2 mL/well and incubated at 37°C overnight. After 48-h transduction, the expression of related genes in the hippocampal neurons after different treatment was determined by reverse transcription quantitative polymerase chain reaction (RT-qPCR).

### Modified Neurological Severity Score

The neurological deficit score was assessed 72 h after ICH modeling using the modified Neurological Severity Score (mNSS) system. This system was used to assess abnormal movements and motor, sensory, and reflex dysfunctions. The scoring included the following: severe injury (10–14 points), moderate injury (5–9 points), and mild injury (1–4 points) (Li et al., 2000; Han et al., 2018). The mNSS was conducted three times independently by three evaluators blinded to the treatment.

### Cerebral Water Content Measurement

Cerebral water content was evaluated by drying wet method. Rats were deeply anesthetized with pentobarbital sodium 72 h after ICH modeling and euthanatized. Rat brains were collected and divided into four parts: left hemisphere, right hemisphere, cerebellum, and brain stem. Then, an electronic analytical balance (Sartorius BS 210 S, Göttingen, Germany) was employed to weigh the water content of each part. Tissues were immediately weighed to obtain the wet weight (WW) and then dried at 95–100°C for 24 h to obtain the dry weight (DW). The cerebral water content (%) = (WW – DW)/WW.

### Blood–Brain Barrier Detection

The quantitative analysis of Evans blue (EB) permeability was detected by EB extravasation using a spectrophotometer. EB solution 2% (wt/vol) was injected into the right tail vein of rats at 4 mL/kg 3 h before euthanasia. Rats were perfused extravascularly with 50 mL of frozen 0.1 M PBS (pH 7.4) under anesthesia to remove the EB dye in the cerebral circulation, and then the brain was taken out. Brain samples of left hemisphere and right hemisphere were divided immediately. The tissue samples were then incubated in 2 mL of 50% trichloroacetic acid. After homogenization and centrifugation at 10,000 × *g* for 20 min, the supernatant (1 mL) was diluted with ethanol (1:3), and the fluorescence intensity at an excitation wavelength of 620 nm and an emission wavelength of 680 nm were measured with an automatic microplate reader. The results were expressed as EB dye (mg)/tissues (g).

### Immunohistochemistry Staining

Paraffin-embedded brain sections with a thickness of 4 μm were prepared as described above, and antigens were recycled by heat-treating on sections in EDTA buffer solution in a microwave oven for 21 min. The endogenous peroxidase activity was inactivated by 0.3% H_2_O_2_ for 10 min. After blocking with 5% bovine serum albumin (BSA) for 20 min, the sections were incubated with primary antibodies (Abcam, Cambridge, UK) to Nrf2 (ab31163, 1:100) and HO-1 (Ab189491, 1:250) at 4°C overnight. Afterward, the sections were incubated with secondary antibodies (1:500, Abcam) to biotinylated goat anti-rat immunoglobulin G (IgG, ab190475) and goat anti-rabbit IgG (ab150077) for 20 min. Finally, the immune response was detected by 3,3-diaminobenzidine staining, and the sections were counter-stained with hematoxylin. The image was acquired using a microscope (Leica-DM2500, Wetzlar, German). All sections were evaluated by two independent pathologists. According to the staining intensity of Nrf2 and HO-1, each sample was scored (0: negative, 1: weak, 2: medium, 3: strong), and the percentage of positive cells was assessed (1: 0–25%, 2: 26–50%, 3: 51–75%, 4: >75%). The immunohistochemistry (IHC) score was calculated by multiplying the staining intensity score and the percentage score. The fluorescence intensity was analyzed using the ImageJ program (National Institutes of Health, Bethesda, MD, USA). The number of immunopositive cells in the perihematomal region was counted in a blinded manner and was expressed as number/0.1-mm^2^ areas. In view of the fact that our research focus was the hippocampus, the regions selected in the IHC experiment to determine the fluorescence intensity mentioned in this article were all hippocampus.

### Immunofluorescence and TUNEL Staining

Sections were blocked in 10% BSA at 25°C for 1 h. Then, these sections were incubated with primary antibodies: mouse anti-KLF6 (sc-365633, 1:200; Santa Cruz Biotechnology, Santa Cruz, CA, USA), rabbit anti-SIRT5 (AF2791, 1:200; Beyotime, Shanghai, China), rabbit anti-ionized calcium-binding adaptor molecule 1 (Iba1, ab178846, 1:500, Abcam), rabbit anti–glial fibrillary acidic protein (GFAP, ab33922, 1:500, Abcam), rabbit anti-NeuN (ab177487, 1:300, Abcam), and rabbit anti-myeloperoxidase (MPO, ab208670, 1:100, Abcam) at 4°C for 12 to 16 h. After incubation, these sections were incubated with Alexa Fluor 647 donkey anti-goat IgG (Thermo Fisher Scientific Inc., Waltham, MA, USA), Alexa Fluor 488 donkey anti-rat IgG (Thermo Fisher Scientific), and CY3 donkey anti-rabbit IgG (Jackson Immunoresearch Laboratories, West Grove, PA, USA) at 25°C for 1 h, and then stained with DAPI (10 μg/mL, Sigma–Aldrich, St. Louis, MO, USA) at 25°C for 15 min. Sections were then costained with transferase-mediated TUNEL (Cell Death Detection Kit, Roche, Basel, Switzerland) and rabbit anti-NeuN (1:200, Abcam), and the percentage of neuronal apoptosis was analyzed separately. All sections were observed under an Olympus BX51 fluorescence microscope (Olympus, Tokyo, Japan) or a laser scanning confocal microscope (FV500, Olympus). Five hematoma boundary regions were selected in each section, and four parts were selected in each animal for analysis. MPO^+^ and Iba1^+^ neurons were counted. Total number of double-positive cells of TUNEL and NeuN in five areas near the injury area was counted using ImageJ (National Institutes of Health), followed by counting of double-positive cells of Iba1 and KLF6, GFAP and KLF6, NeuN and KLF6, and SIRT5 and NeuN.

### Western Blot Analysis

Total proteins were extracted from tissues or neurons using high-efficiency radioimmunoprecipitation assay (RIPA) lysis buffer (C0481, Sigma–Aldrich) strictly following the manufacturer's instructions. After lysis at 4°C for 15 min, tissues or cells were centrifuged at 10,000 g for 15 min, and the supernatant was extracted. The protein concentration of each sample was determined using a BCA kit (23227, Thermo Fisher Scientific). Subsequent to quantification according to different concentrations, the proteins were separated by sodium dodecyl sulfate–polyacrylamide gel electrophoresis and transferred to a polyvinylidene fluoride membrane, which was blocked with 5% BSA at room temperature for 1 h. Then the membrane was incubated primary rabbit antibodies to KLF6 (PA5-79560, 1:1,000, Invitrogen, Carlsbad, CA, USA), Nrf2 (ab137550, 1:2,000, Abcam), HO-1 (ab68477, 1:10,000, Abcam), SIRT5 (ab259967, 1:1,000, Abcam), and glyceraldehyde-3-phosphate dehydrogenase (GAPDH) (ab8245, 1:1,000, Abcam) overnight. On the second day, the membrane was incubated with peroxidase horseradish–labeled goat anti-rabbit IgG (1:20,000, ab205718, Abcam) at room temperature for 1.5 h. After incubation, the membrane was exposed to developing solution (NCI4106, Pierce, Rockford, IL, USA). Protein quantitative analysis was conducted using ImageJ 1.48u software (Bio-Rad, Hercules, CA, USA). The relative protein level was determined by using the ratio of the gray value of each protein to that of the internal reference GAPDH.

### RT-qPCR

Total RNA was extracted from hippocampal tissue and neuron samples using a Trizol kit (Invitrogen). The quality and concentration of RNA were determined by a UV-Vis spectrophotometry (ND-1000, Nanodrop). Total RNA was extracted using an RNeasy Mini Kit (Qiagen, Valencia, CA, USA). Reverse transcription was conducted using a reverse transcription kit (RR047A, Takara, Tokyo, Japan) to obtain cDNA according to the manual. With cDNA as the template, fluorescent quantitative PCR operation was conducted following the instructions of the instructions of SYBR® Premix^Ex^ Taq™ II (Perfect Real Time) kit (DRR081, Takara) on a real-time fluorescent quantitative PCR machine (ABI 7500, ABI, Foster City, CA, USA). The mRNA level of GAPDH was used as an internal control to normalize the results (Supplementary Table 1). 2^−Δ*ΔCt*^ represented the multiple relationship between the target gene expression of the experimental group and the control group.

### Determination of Malondialdehyde Content, Superoxide Dismutase, and Glutathione Peroxidase Activity

Rat brains were perfused with PBS 72 h after ICH modeling, and then the ipsilateral cortex was homogenized to detect the malondialdehyde (MDA) level and the activity of oxidative stress–related enzymes [superoxide dismutase (SOD) and glutathione peroxidase (GSH-Px)]. According to the manufacturer's instructions, MDA level was tested by the reaction of MDA and thiobarbituric acid under acidic conditions at high temperature, and then the optical density (OD) value was measured. SOD activity was detected by WST-1 method according to the manufacturer's instructions. GSH-Px activity was detected by the decrease of nicotinamide adenine dinucleotide phosphate in the reaction system according to the manufacturer's instructions.

### Determination of 3-Nitrotyrosine and 8-Hydroxy-2′-Deoxyguanosine Content

The levels of 3-nitrotyrosine (3-NT) and 8-hydroxy-2′-deoxyguanosine (8-OHdG) in the hippocampus of bleeding 72 h after ICH modeling were measured using an enzyme-linked immunosorbent assay (ELISA) kit. Samples were incubated with the primary antibody overnight at 4°C, with the secondary antibody at room temperature for 1 h, and with the substrate solution at room temperature for 15 min in the dark. The reactions were stopped by adding corresponding solutions to each sample. The OD value at 450 nm was measured using a SpectraMax M2 spectrometer (Molecular Devices, Sunnyvale, CA, USA), and the protein level was calculated.

### Dual Luciferase Reporter Gene Assay

The 293T cells were seeded in a six-well plate at a density of 5 × 10^5^ cells/well before transduction. Cells were transduced when the cell density reached about 60%. pRL constructs containing the Renilla luciferase reporter gene were cotransduced with pGL3-KLF6 vector, pcDNA3.1 control, and pcDNA3.1-KLF6 expression vector into 293T cells. After 48 h, the luciferase activity was measured on the dual luciferase reporter gene assay system (Promega, Madison, WI, USA). The luciferase activity = relative luminescence unit (RLU) value of the firefly luciferase/RLU value of Renilla luciferase.

### Chromatin Immunoprecipitation Assay

Process of chromatin immunoprecipitation (ChIP) assay was as follows: (1) hippocampal neuron collection. The 2 × 100-mm cells in culture dishes were fixed with 1% formaldehyde, which was terminated with 0.125 M glycine. (2) Ultrasonic disruption. Neurons were resuspended in RIPA buffer (P0013B, Beyotime). The genomic DNA of adipose tissues was broken into 200–700-bp size fragments using a diagenode ultrasonic disintegrator. (3) ChIP: the DNA concentration of samples was measured and divided into input, KLF6 and IgG groups. Dynabeads™ Protein G (Thermo Fisher Scientific) and corresponding antibodies KLF6 (sc-365633, 1:200, Santa Cruz Biotechnology) and IgG (ab190475, 1:200, Abcam) were added into KLF6 and IgG groups, respectively, and incubated with immunoprecipitated chromatin at 4°C overnight. (4) Uncrosslinking and DNA extraction. The DNA sample incubated with antibody was eluted, and a DNA extraction kit was used to extract DNA. DNA was dissolved and stored at −20°C for later use. (5) RT-qPCR detection of immunoprecipitated DNA fragments. RT-qPCR was used to detect the degree of DNA enrichment by immunoprecipitation. The primer sequences used are presented in Supplementary Table 2.

### Fluoro-Jade C Staining

Fluoro-Jade C (FJC), a polyanionic fluorescein derivative, was used in this study to detect neuronal degradation. Brain tissue sections were deparaffinized, dehydrated, and then incubated in 0.06% potassium permanganate solution (Sigma–Aldrich) for 10 min. Next, sections were rinsed in deionized water, immersed in FJC working solution (0.1% acetic acid) for 20 min, and dried in an incubator. Finally, the sections were washed in xylene and fixed by anhydrous, low-fluorescence, styrene-based mounting medium (DPX, Sigma–Aldrich). FJC-positive cells were observed by a fluorescence microscope (Olympus) and counted by technicians who did not know the experimental conditions.

### MitoSOX Staining

Primary hippocampal neurons were exposed to OxyHb (20 μM) to simulate the ICH modeling *in vitro*. After 24 h, a stock solution of 5-mM MitoSOX reagent was prepared. Then, 13 mL of dimethyl sulfoxide solution was added to a bottle of MitoSOX Red MitoSOX indicator (Thermo Fisher Scientific) containing 50-mg content. Then, the 5-mM MitoSOX reagent working solution was prepared by diluting the 5-mM MitoSOX reagent stock solution with PBS. Neurons were all covered with 5-mM MitoSOX reagent working solution and incubated for 10 min at 37°C in the dark. Then the neurons were loaded into PBS for analysis and imaging.

### Dihydroethidium Staining

Dihydroethidium (DHE) staining was performed to detect superoxide anions, which reflected the level of oxidative stress in tissues. Rats were perfused with endocardium under anesthesia 72 h after ICH modeling. Samples were immediately frozen at −80°C and cut into 15-mm-thick coronal brain sections by a freezing microtome (CM 1950, Leica). The slices at 0.58 mm posterior to bregma were stained with DHE for 30 min. Sections were stained with DHE for 30 min. Then, the ventral side of the left hemisphere was observed with a laser scanning confocal microscope (A1 Si, Nikon, Tokyo, Japan). The representative image was taken from sections of 2 mm behind the bregma.

### JC-1 Staining

The changes in neuronal mitochondrial membrane potential were detected using a mitochondrial membrane potential measurement kit, and JC-1 staining was used as an indicator of mitochondrial damage (Beyotime), which were both conducted in accordance with the manufacturer's procedures. The pretreated neurons were incubated with 1 mL of JC-1 working solution per sample for 20 min at 37°C. The neurons were then washed twice with JC-1 staining buffer. After being added with DAPI (DAPI Fluoromount-G, SouthernBiotech, Birmingham, AL, USA), neurons were observed under a fluorescence microscope.

### Flow Cytometry

After 48 h of transduction, cells were disrupted with 0.25% pancreatin without EDTA (YB15050057, Yubo Biotech Co., Ltd., Shanghai, China), collected in a flow tube, and centrifuged, followed by removal of the supernatant. Cells were centrifuged, and the supernatant was discarded. According to the instructions of the annexin-V–fluorescein isothiocyanate (FITC) cell apoptosis detection kit (K201-100, Biovision, Milpitas, CA, USA), the annexin-V–FITC, propidium iodide (PI), and 4-(2-hydroxyethyl)-1-piperazineethanesulfonic acid (HEPES) buffer solutions were made into annexin-V–FITC/PI dye solution at a ratio of 1:2:50. Then, 1 × 10^6^ cells were resuspended in 100 μL dye solution, shaken, and mixed with 1 mL HEPES buffer solution (PB180325, Procell, Wuhan, Hubei, China) after incubation at room temperature for 15 min. Bandpass filters of 525 and 620 nm were excited by a wavelength of 488 nm to detect FITC and PI fluorescence, respectively, for assessment of neuronal apoptosis.

### Statistical Analysis

Statistical analysis was performed using the SPSS 21.0 software (IBM Corp. Armonk, NY, USA). The measurement data were expressed as mean ± standard deviation. Data between two groups were analyzed using unpaired *t*-test, and those among multiple groups were tested by one-way analysis of variance (ANOVA) followed by Dunnett *post-hoc* test. mNSS scores were compared using non-parametric test. A *p* < 0.05 was considered to be statistically significant.

## Results

### KLF6 Was Highly Expressed in the Hippocampal Tissues of ICH Rats

**Figure 1:**
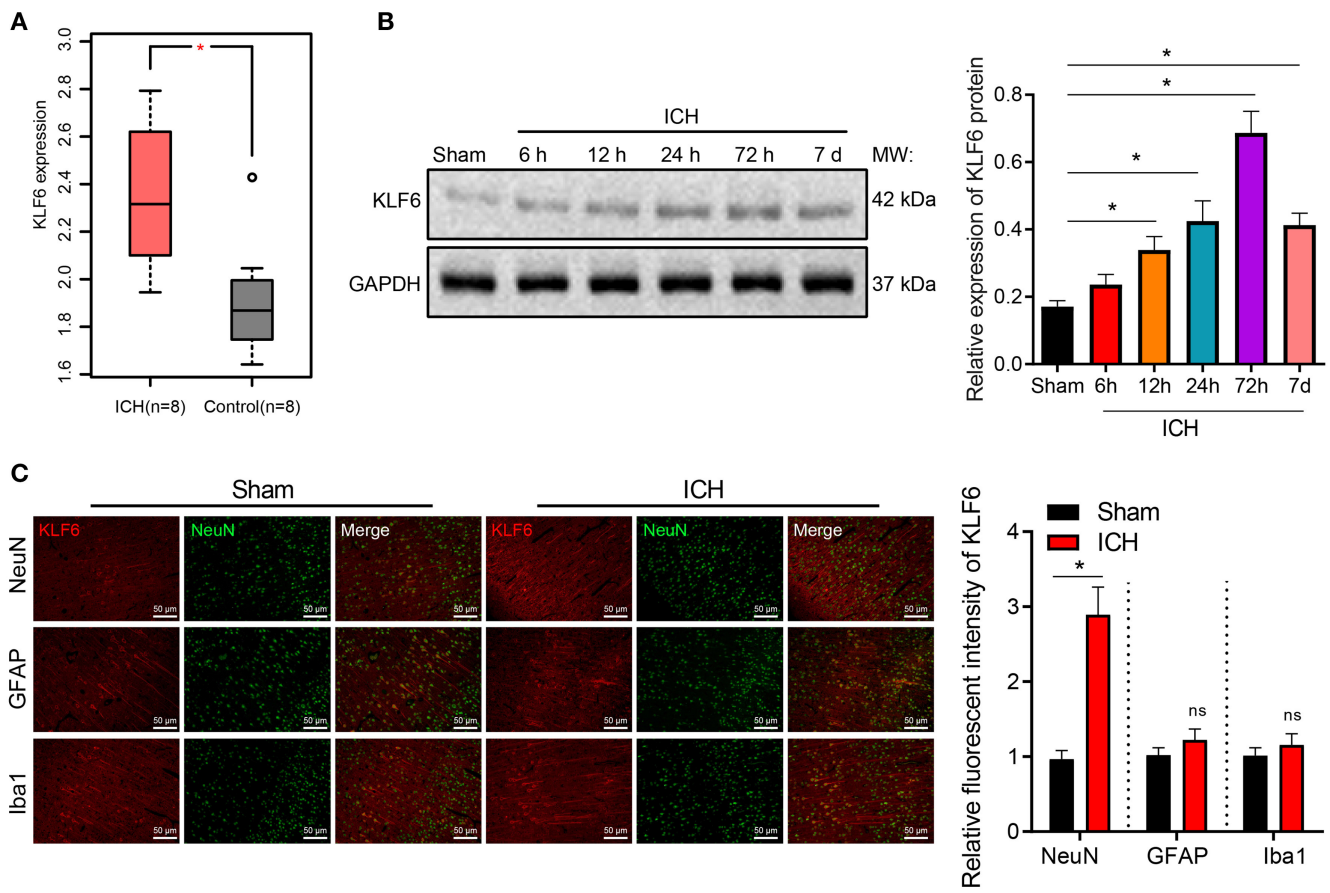
The expression of KLF6 is high in hippocampal neurons of ICH model rats. **(A)** The expression of KLF6 in the ICH-related microarray GSE149317. The box diagram shows the expression of KLF6 gene; the red one indicates the ICH group, and the gray one indicates the normal group. **(B)** KLF6 expression in rats at different time points (6, 12, 24, 72 h, 7 days) after ICH modeling determined by Western blot analysis. **(C)** KLF6 expression and location in different neurons detected by immunofluorescence staining. *p < 0.05 compared with sham-operated rats. The measurement data were expressed as mean ± standard deviation. Comparisons between two groups were analyzed using unpaired *t*-test, and comparisons among multiple groups were tested by one-way ANOVA followed by Dunnett *post-hoc* test. *n* = 6 rats/group.

ICH-related microarray GSE149317 was obtained through GEO database (https://www.ncbi.nlm.nih.gov/geo/), which included eight normal brain tissues and eight ICH brain tissues. The differential analysis by edgeR software package of R language revealed that KLF6 was upregulated in the brain tissues of ICH patients (Figure 1A).

**Figure 2:**
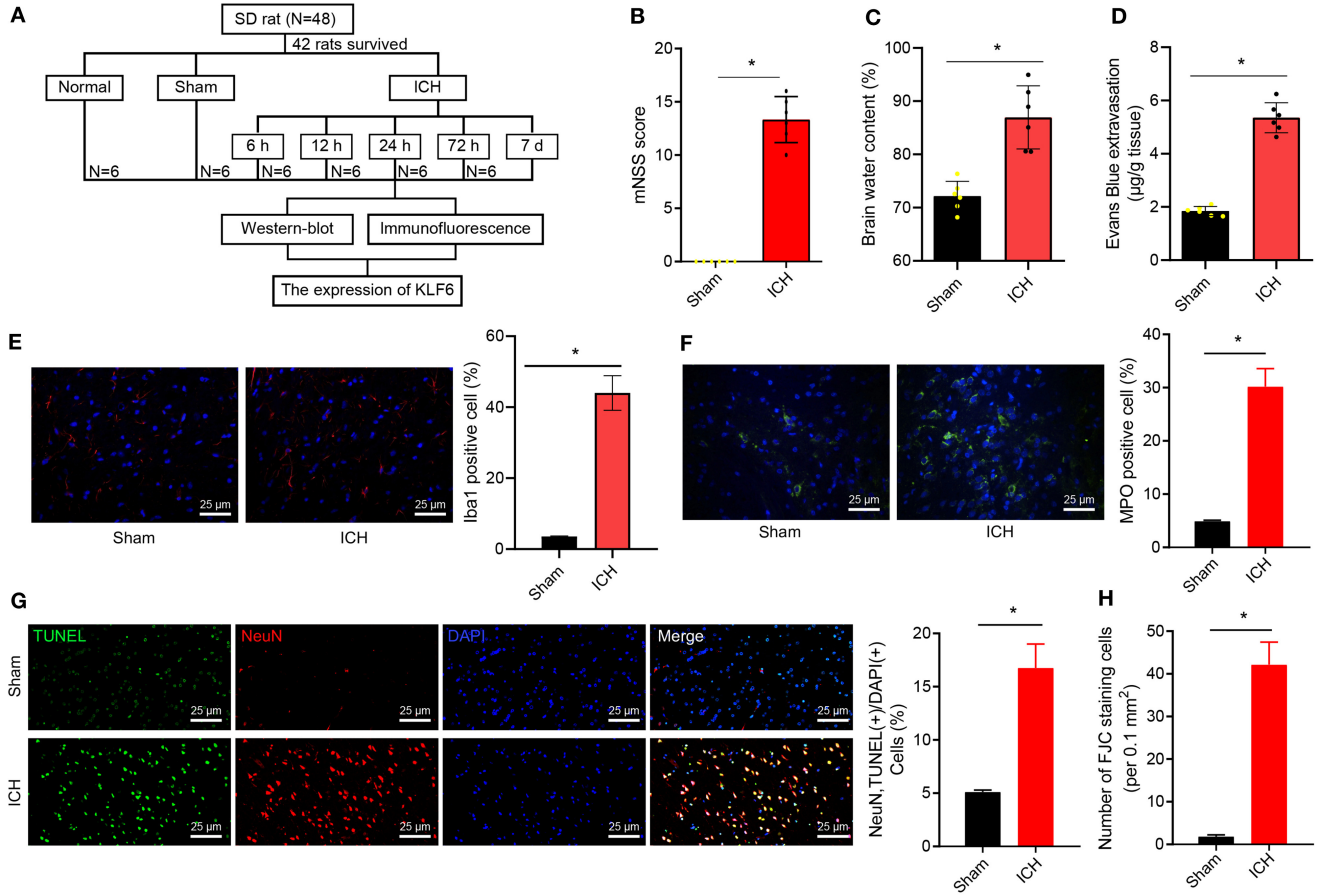
Successful establishment of ICH rat models. **(A)** ICH model establishment. **(B)** mNSS scoring of rats 72 h after ICH modeling. The scoring includes severe injury (10–14 points), moderate injury (5–9 points), and mild injury (1–4 points). **(C)** Water content in brain tissues of rats 72 h after ICH modeling. **(D)** The penetration of Evans blue in rat brain tissues 72 h after ICH modeling. **(E)** The activation of microglia 72 h after ICH modeling detected by immunofluorescence staining (Iba1^+^ cells were quantified). **(F)** The activation of neutrophils was detected by immunofluorescence staining (MPO^+^ cells were quantified). **(G)** The apoptosis of hippocampal neurons 72 h after ICH modeling detected by TUNEL staining (NeuN^+^ TUNEL^+^ cells were quantified). **(H)** The quantitative analysis of degeneration of hippocampal neurons in the cerebral cortex 72 h after ICH modeling detected by FJC staining. *p < 0.05 compared with sham-operated rats. *n* = 6 rats/group. The measurement data were expressed as mean ± standard deviation. Comparisons between two groups were analyzed using unpaired *t*-test, and comparisons among multiple groups were tested by one-way ANOVA followed by Dunnett *post-hoc* test.

To further explore the mechanism of KLF6 in the pathogenesis of ICH, ICH rat models were initially induced by injecting autologous blood into the right basal ganglia (Figure 2A). Then, the coronal section of the hippocampus of ICH rats 72 h after modeling was observed, and the neurological dysfunction of rats 72 h after modeling was detected by mNSS score. The results demonstrated that compared with sham-operated rats, the mNSS score of ICH rats increased notably (Figure 2B). Water content of brain tissues of ICH rats increased compared with sham-operated rats (Figure 2C).

EB staining was used to detect the permeability of the blood–brain barrier in rats. The results uncovered that the EB permeability of the brain tissues of ICH rats was notably augmented compared with that of sham-operated rats (Figure 2D). Iba1^+^ and MPO^+^ cells were detected by immunofluorescence staining to evaluate the activation of inflammatory cells (microglia and neutrophils) in the hippocampus. The results showed that the Iba1^+^ and MPO^+^ cells were notably increased in hippocampal tissues of ICH rats compared to those of sham-operated rats (Figures 2E,F). For TUNEL staining, the rate of NeuN^+^ TUNEL^+^ cells in ICH rats was elevated in contrast to sham-operated rats (Figure 2G). FJC staining was used to detect the degeneration of hippocampal neurons. The results showed that the number of FJC-positive cells in the hippocampus of the ICH rats was higher than that of sham-operated rats (Figure 2H).

Furthermore, Western blot analysis at 6, 12, 24, 72 h, and 7 days after modeling depicted that in comparison with sham-operated rats, the expression of KLF6 in the ICH rats increased with the increase of bleeding time, reached a peak at 72 h, and then diminished (Figure 1B). Subsequently, the colocalization of KLF6 with Iba1, NeuN, and GFAP in the hippocampus of ICH rats was detected by immunofluorescence staining. The results showed that the fluorescence intensity of KLF6 in hippocampal neurons of ICH rats was more notable than that in microglia and astrocytes (Figure 1C).

To sum up, KLF6 was up-regulated in the hippocampal tissues of ICH rats, its expression reached the highest at 72 h after ICH modeling, and KLF6 was abundantly expressed in hippocampal neurons.

### KLF6 Silencing Relieved Neurological Dysfunction of ICH Rats

**Figure 3:**
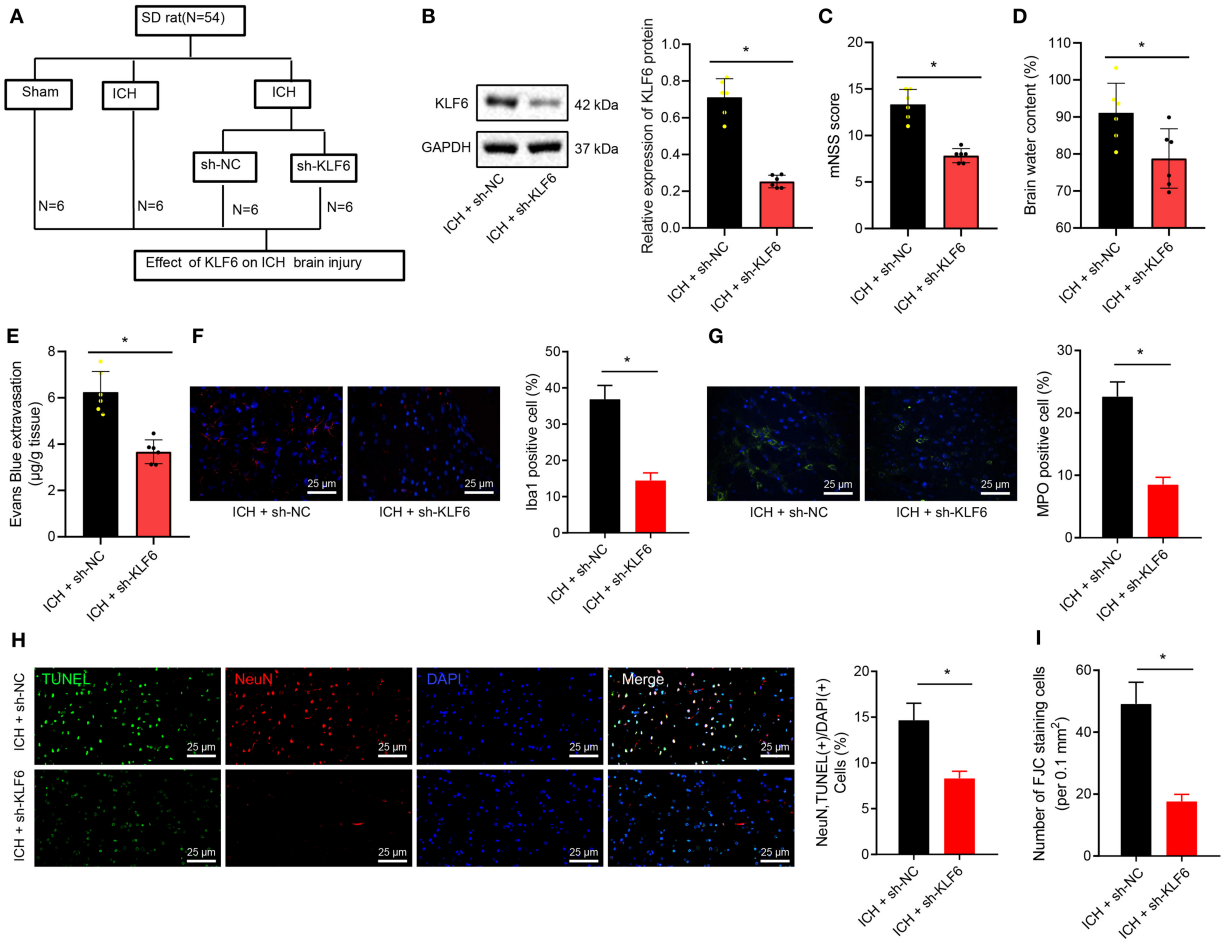
KLF6 silencing attenuates neurological dysfunction and hippocampal neuronal apoptosis and degeneration in ICH rats. **(A)** Establishment of ICH model with KLF6 silencing (*n* = 6). **(B)** The level of KLF6 in the hippocampal tissues of three paired ICH rats 72 h after modeling and infection with sh-KLF6. **(C)** The mNSS score of ICH rats 72 h after modeling and infection with sh-KLF6. **(D)** The cerebral water content changes in brain tissues of ICH rats 72 h after modeling and infection with sh-KLF6. **(E)** Evans blue permeation in brain tissues of ICH rats 72 h after modeling and infection with sh-KLF6. **(F)** The activation of microglia in ICH rats 72 h after modeling and infection with sh-KLF6 (Iba1^+^ cells was quantified). **(G)** The activation of neutrophils in ICH rats 72 h after modeling and infection with sh-KLF6 (MPO^+^ cells were quantified). **(H)** The apoptosis of hippocampal neurons in ICH rats 72 h after modeling and infection with sh-KLF6 (NeuN^+^ TUNEL^+^ cells was quantified). **(I)** The quantitative analysis of degeneration of hippocampal neurons in ICH rats 72 h after modeling and infection with sh-KLF6 as detected by FJC staining. *n* = 6 rats/group *p < 0.05 compared with ICH rats infected with sh-NC. The measurement data were expressed as mean ± standard deviation. Comparisons between two groups were analyzed using unpaired *t*-test, and comparisons among multiple groups were tested by one-way ANOVA followed by Dunnett *post-hoc* test.

Furthermore, KLF6 was silenced in ICH rats by injecting sh-KLF6 into the lateral ventricle of ICH rats (Figure 3A) to explore the mechanism of KLF6 in ICH neurological dysfunction. Western blot analysis results displayed that the expression of KLF6 in the hippocampal tissues of ICH rats infected with sh-KLF6 was reduced compared with that of ICH rats infected with sh-NC (Figure 3B). The mNSS score of the ICH rats infected with sh-KLF6 was lowered (Figure 3C). The water content of brain tissues of ICH rats infected with sh-KLF6 was diminished (Figure 3D).

For EB staining, the permeability of the blood–brain barrier in brain tissues of ICH rats was reduced after the infection of sh-KLF6 (Figure 3E). The results of immunofluorescence manifested that the number of Iba1^+^ and MPO^+^ cells in the hippocampal tissues of ICH rats infected with sh-KLF6 was notably reduced (Figures 3F,G). According to results of TUNEL staining, the apoptosis of neurons in the hippocampal tissue of ICH rats was declined by silencing KLF6 (Figure 3H). Based on the results of FJC staining, sh-KLF6 treatment caused reduction of FJB-positive cells in the hippocampus of ICH rats (Figure 3I).

Together, downregulation of KLF6 reduced the blood–brain barrier permeability and inflammatory cell activation and inhibited the degeneration and apoptosis of neurons in ICH rats, thereby alleviating brain tissue damage.

### KLF6 Silencing Inhibited Oxidative Stress in Hippocampus of ICH Rats

**Figure 4:**
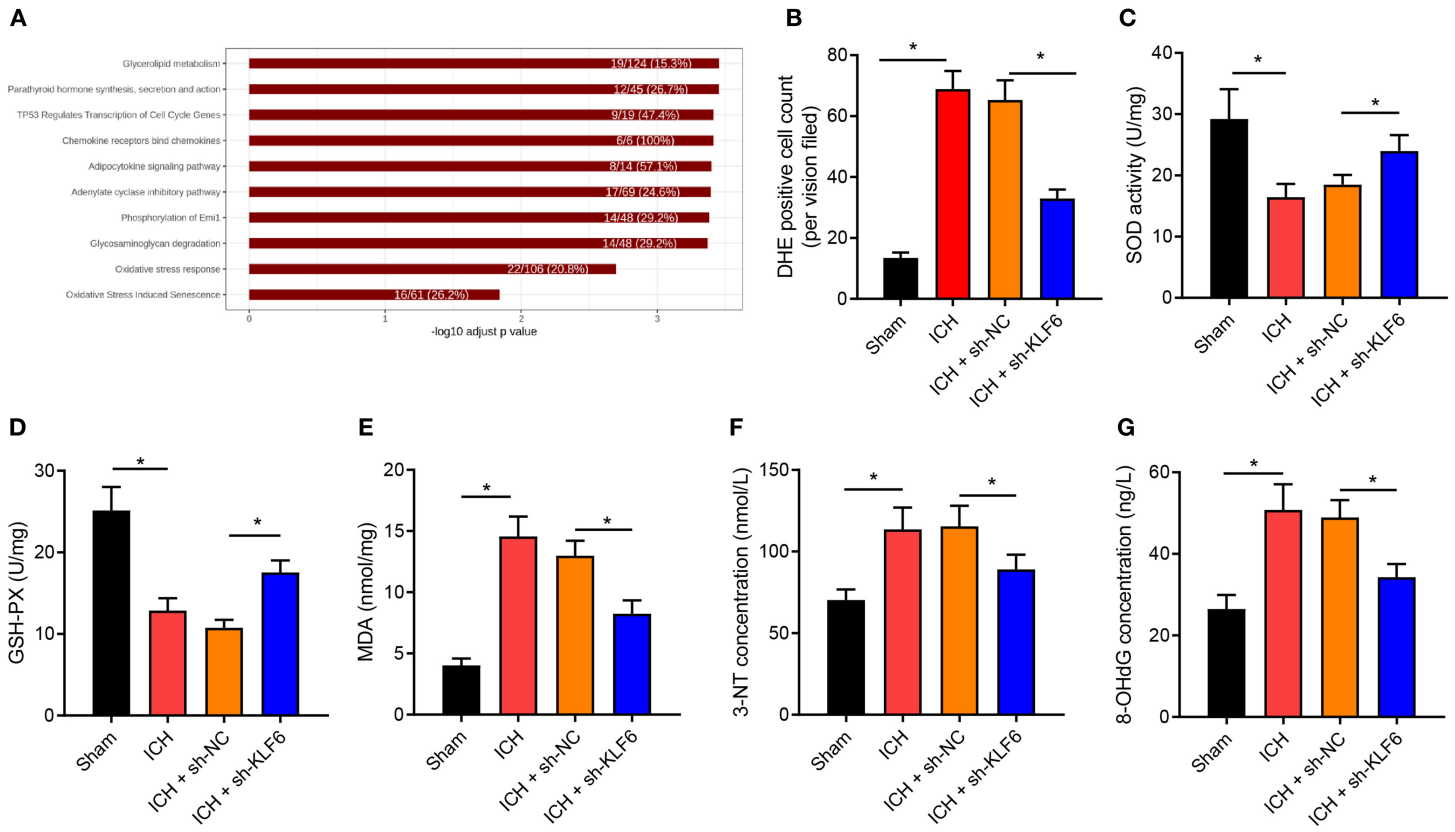
KLF6 silencing represses oxidative stress in the hippocampus of ICH rats. **(A)** Kobas v3.0 enrichment analysis of KLF6 regulation pathway in ICH-related microarray GSE149317. **(B)** Reactive oxygen in the hippocampus of rats 72 h after ICH modeling and infection with sh-KLF6 detected by DHE staining. **(C)** SOD activity in hippocampus of ICH rats after different infection. **(D)** GSH-Px activity in hippocampus of ICH rats after different infection. **(E)** MDA level in hippocampus of ICH rats after different infection. **(F)** 3-NT concentration changes in hippocampus of ICH rats after different infection. **(G)** 8-OHdG concentration changes in hippocampus of ICH rats after different infection. *n* = 6 rats/group. *p < 0.05. The measurement data were expressed as mean ± standard deviation. Comparisons between two groups were analyzed using unpaired *t*-test, and comparisons among multiple groups were tested by one-way ANOVA followed by Dunnett *post-hoc* test.

DEGs in the ICH-related microarray GSE149317 were analyzed, and correlation analysis with KLF6 was performed. Genes related to KLF6 expression were selected, and enrichment analysis was conducted using Kobas v3.0. The results revealed that KLF6 may be involved in the process of ICH by regulating oxidative stress (Figure 4A). To further explore whether KLF6 was involved in oxidative stress in ICH, ROS fluorescent probe–DHE staining was used to detect the level of oxidative stress in the hippocampus of rats, and it was observed that compared to sham-operated rats, the number of DHE-positive cells in the hippocampus of the ICH rats increased notably, and it was reduced after ICH rats were infected with sh-KLF6 (Figure 4B).

Further, the expression of oxidative stress–related enzyme SOD and GSH-Px activity and MDA content were determined, and the results exhibited that the SOD and GSH-Px enzyme activities were diminished, and the MDA content was augmented in the hippocampus of ICH rats vs. sham-operated rats, which was reversed by sh-KLF6 treatment (Figures 4C–E). ELISA data described that the content of 3-NT and 8-OHdG in the hippocampus of ICH rats increased in contrast to sham-operated rats, but was diminished in ICH rats after silencing sh-KLF6 (Figures 4F,G).

Together, the level of oxidative stress increased remarkably in ICH rats, and KLF6 silencing inhibited the oxidative stress caused by ICH in rats.

### KLF6 Silencing Inhibited OxyHb-Induced Oxidative Stress in Hippocampal Neuron

**Figure 5:**
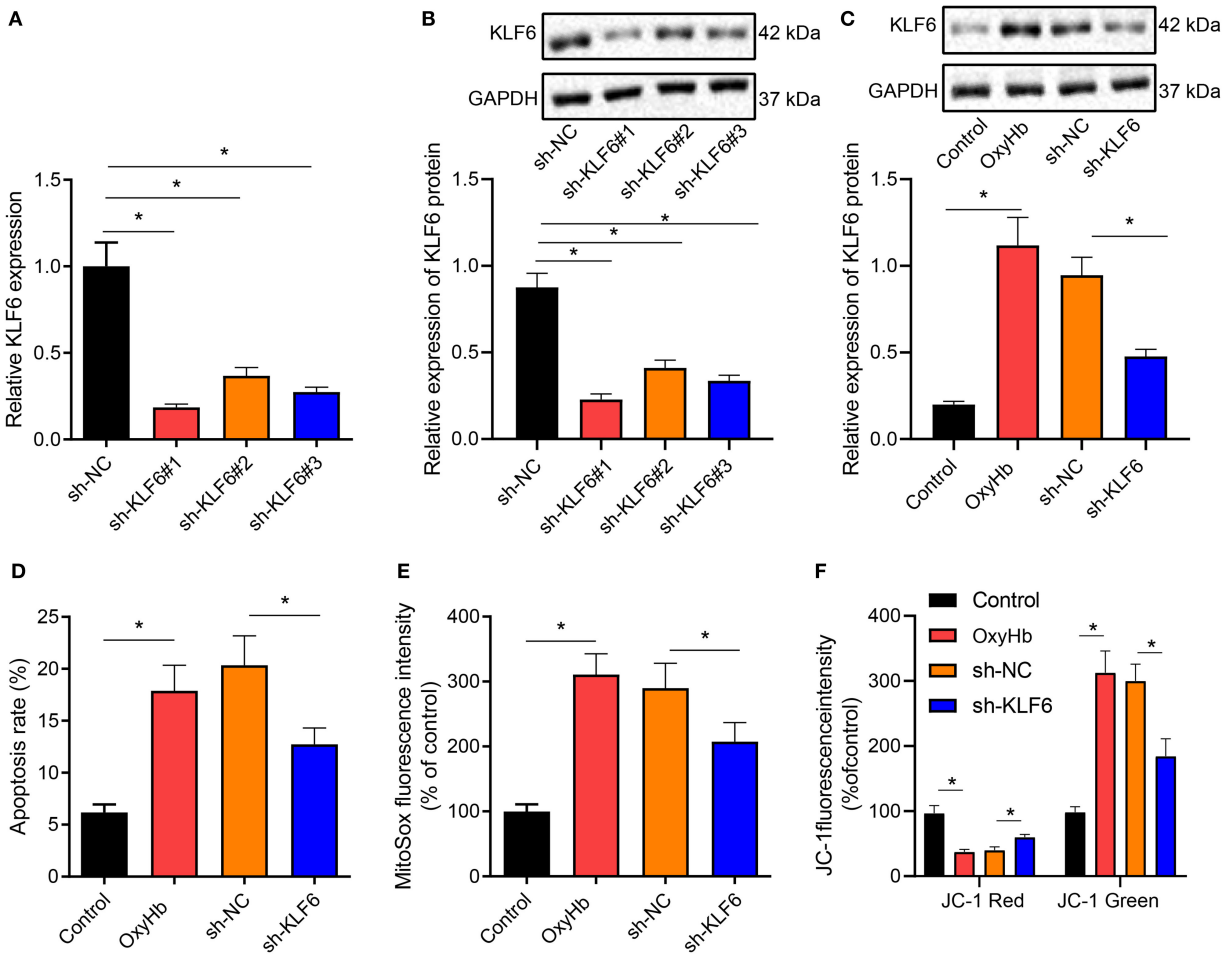
KLF6 silencing depresses OxyHb-induced mitochondrial ROS and mitochondrial damage in hippocampal neurons. **(A)** The level of KLF6 in hippocampal neurons after KLF6 silencing determined by RT-qPCR. **(B)** The level of KLF6 in hippocampal neurons after KLF6 silencing determined by Western blot analysis. **(C)** The expression of KLF6 in hippocampal neurons after 20 μM OxyHb induction and KLF6 silencing determined by Western blot analysis. **(D)** The effect of KLF6 silencing on OxyHb-induced hippocampal neuronal apoptosis determined by flow cytometry. **(E)** The changes of mitochondrial ROS in neurons after 20 μM OxyHb induction and KLF6 silencing detected by MitoSOX fluorescence staining. **(F)** Mitochondrial membrane potential changes after 20 μM OxyHb induction and KLF6 silencing determined by JC-1 staining. *p < 0.05. The measurement data were expressed as mean ± standard deviation. Comparisons between two groups were analyzed using unpaired *t*-test, and comparisons among multiple groups were tested by one-way ANOVA followed by Dunnett *post-hoc* test. The experiment was repeated three times.

To further verify whether KLF6 was involved in regulating oxidative stress in ICH, hippocampal neurons were exposed to 20 μM OxyHb to induce an *in vitro* cell model of ICH. Then the expression of KLF6 was determined by RT-qPCR and Western blot analysis after silencing KLF6 in primary hippocampal neurons by lentivirus, and it was observed that compared with neurons infected with sh-NC, the expression of KLF6 was notably lowered in neurons infected with sh-KLF6#1, sh-KLF6#2, and sh-KLF6#3, among which sh-KLF6#1 presented the superior efficiency and was thus selected for subsequent experiments (Figures 5A,B).

Hippocampal neurons after the infection of sh-KLF6 were exposed to 20 μM OxyHb. Western blot analysis revealed that OxyHb induction notably promoted the expression of KLF6 in hippocampal neurons, which was negated by further sh-KLF6 treatment (Figure 5C). Also, the apoptosis rate of hippocampal neurons was accelerated by OxyHb induction, which was annulled by silencing KLF6 (Figure 5D). MitoSOX fluorescence staining was performed to detect the changes of mitochondrial ROS in hippocampal neurons. The results manifested that OxyHb promoted the fluorescence intensity of MitoSOX in neurons, which was neutralized by sh-KLF6 (Figure 5E).

Then, changes of cell membrane potential in hippocampal neurons were detected by JC-1 staining. The results demonstrated that OxyHb induced a decrease in red fluorescence intensity and an increase in green fluorescence in hippocampal neurons, indicating that the mitochondrial membrane potential diminished and the mitochondrial permeability transition pore was opened. Moreover, the red intensity increased, the green fluorescence intensity diminished, and the mitochondrial membrane potential increased notably in OxyHb-induced hippocampal neurons by sh-KLF6 treatment (Figure 5F).

Therefore, KLF6 silencing inhibited OxyHb-induced hippocampal neuronal apoptosis and mitochondrial damage.

### SIRT5 Was Poorly Expressed in Hippocampal Tissues of ICH Rats and Was Transcriptionally Inhibited by KLF6

**Figure 6:**
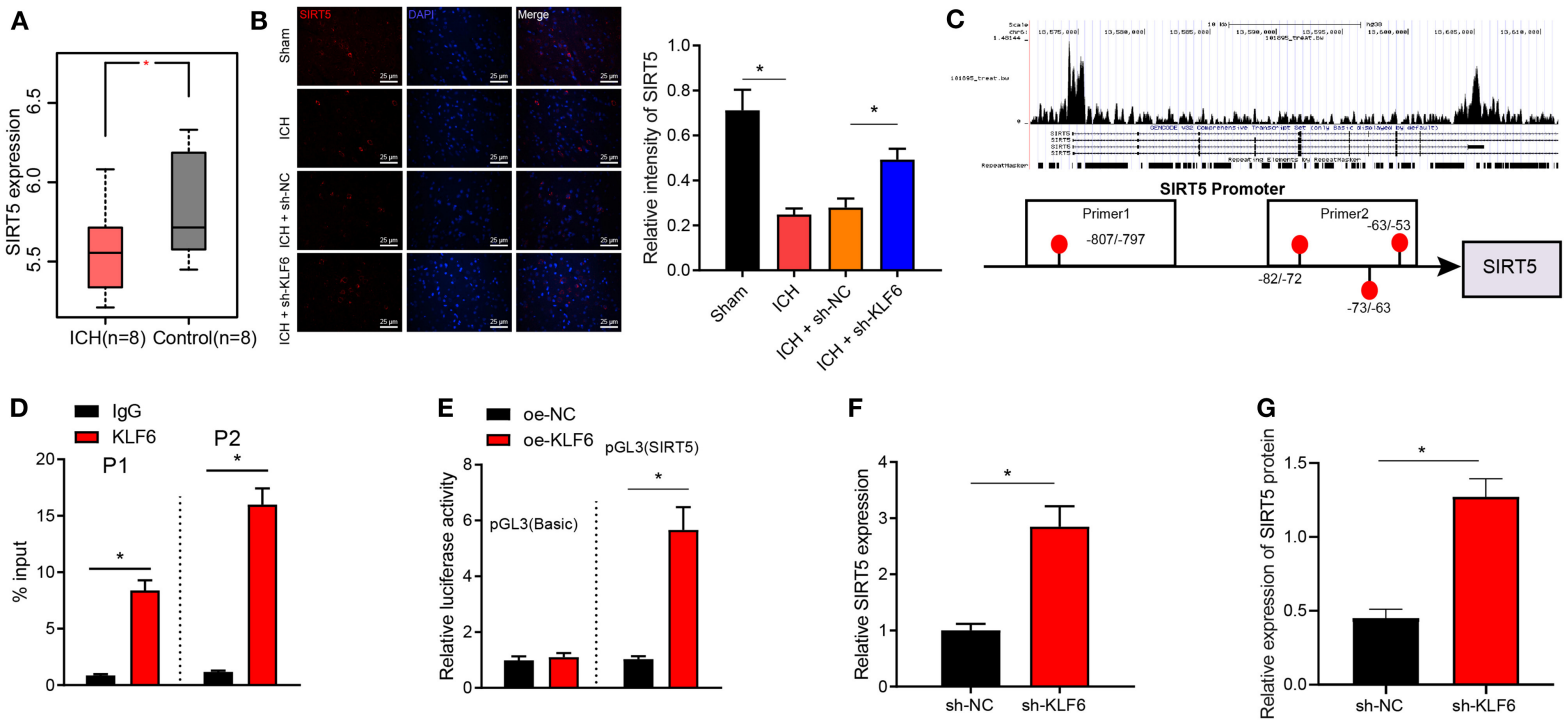
KLF6 binds to SIRT5 promoter to inhibit its transcription. **(A)** The expression of SIRT5 in the ICH-related microarray GSE149317. The box diagram represents the expression of SIRT5 gene. **(B)** SIRT5 colocalization with hippocampal neurons in ICH rats detected by immunofluorescence staining. **(C)** The enrichment of KLF6 in the SIRT5 promoter region detected by CistromeDB database ChIP-Seq data, and the binding site of KLF6 and SIRT5 promoter predicted by JASPAR database. **(D)** The enrichment of KLF6 in SIRT5 promoter −20/−220 (Prime2) and −700/−900 (Prime1) regions determined by ChIP assay. **(E)** The effect of KLF6 overexpression on the luciferase activity of SIRT5 promoter determined by dual luciferase reporter gene assay. **(F)** The level of SIRT5 in hippocampal neurons infected with sh-KLF6 determined by RT-qPCR. **(G)** The level of SIRT5 in hippocampal neurons infected with sh-KLF6 determined by Western blot analysis. *p < 0.05. The measurement data were expressed as mean ± standard deviation. Comparisons between two groups were analyzed using unpaired *t*-test, and comparisons among multiple groups were tested by one-way ANOVA followed by Dunnett *post-hoc* test. The experiment was repeated three times.

Differential analysis of ICH-related microarray GSE149317 was conducted by edgeR software package of R language, which observed that SIRT5 was downregulated in the hippocampal tissues of ICH rats (Figure 6A). Moreover, SIRT5 was downregulated in neurons of ICH rats compared with sham-operated rats but was enhanced in neurons of ICH rats infected with sh-KLF6 (Figure 6B). The peak chart of ChIP-Seq data from CistromeDB database identified that KLF6 was enriched in the promoter region of SIRT5, and it was predicted by the JASPAR database that there were binding sites between KLF6 and SIRT5 promoter (Figure 6C). The enrichment of KLF6 in the promoter region of SIRT5 at −20/−220 bp (Prime2) and −700/−900 bp (Prime1) was evaluated using ChIP assay; it was depicted that compared with IgG, KLF6 was enriched in Prime1 and Prime2, and the degree of enrichment was higher in the Prime2 (Figure 6D). Dual luciferase reporter gene assay was conducted to detect the effect of KLF6 overexpression on the luciferase activity of the SIRT5 Prime2 promoter region. The results identified that KLF6 overexpression significantly enhanced luciferase activity in pGL3-SIRT5 and did not affect the luciferase activity of pGL3-basic plasmids (Figure 6E). The results of RT-qPCR and Western blot analysis demonstrated that the mRNA and protein levels of SIRT5 were elevated in hippocampal neurons infected with sh-KLF6 (Figures 6F,G). In summary, SIRT5 was lowly expressed in ICH hippocampus and was repressed by transcriptionally KLF6.

### KLF6 Silencing Suppressed OxyHb-Induced Oxidative Stress in Hippocampal Neurons Through Inactivation of the Nrf2/HO-1 Signaling Pathway by Upregulating SIRT5

**Figure 7:**
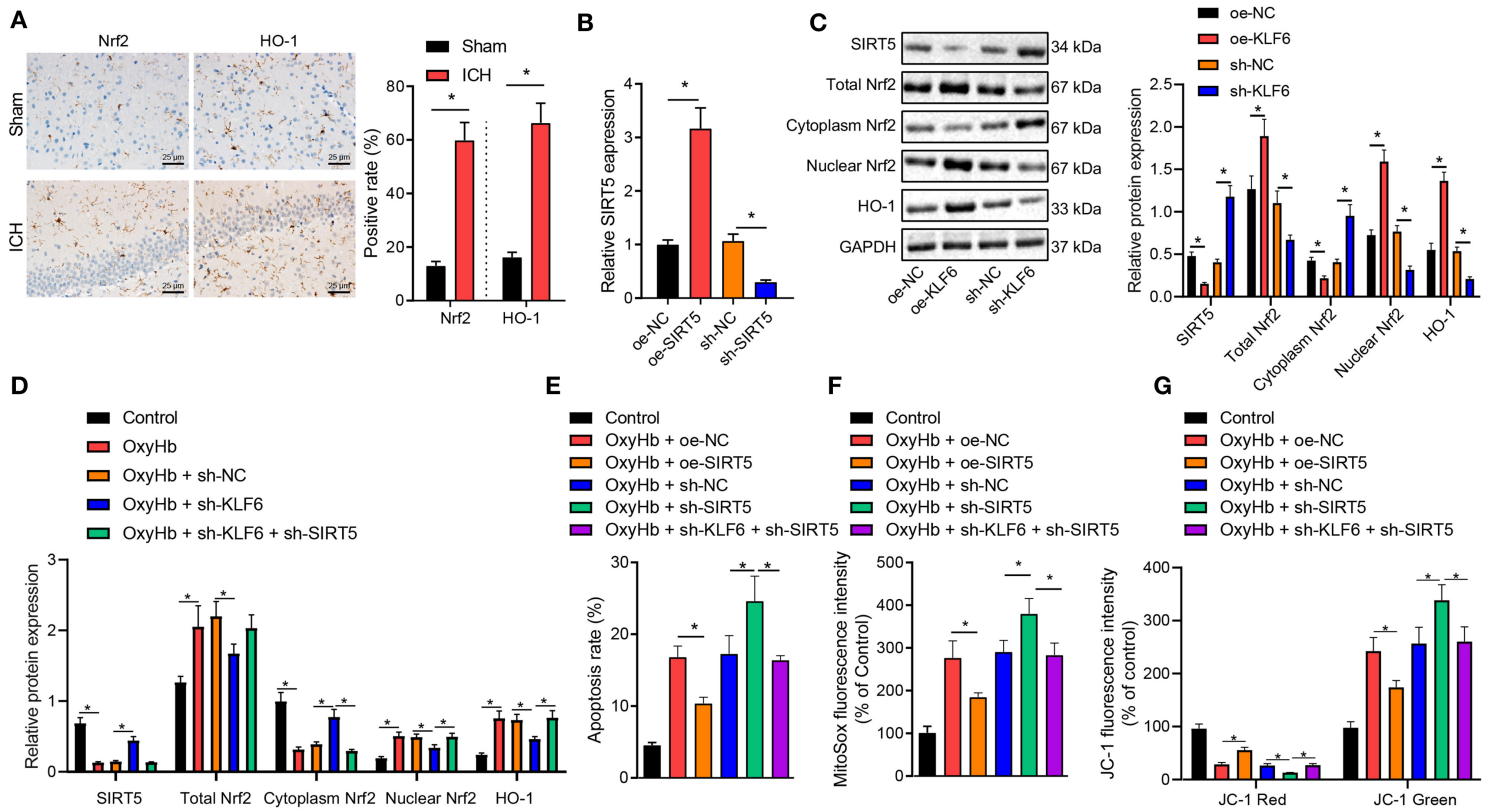
Silencing KLF6 causes inhibition of mitochondrial ROS and mitochondrial damage of OxyHb-induced hippocampal neurons *via* the SIRT5/Nrf2/HO-1 axis. **(A)** The expression of Nrf2 and HO-1 in the hippocampus of ICH rats detected by IHC (*n* = 6). **(B)** The expression of SIRT5 in hippocampal neurons after the silencing and overexpression of SIRT5 determined by RT-qPCR. **(C)** The expression of SIRT5, Nrf2 (total, cytoplasm, and nuclear), and HO-1 in hippocampal neurons after the silencing and overexpression of KLF6 determined by Western blot analysis. **(D)** The expression of Nrf2 (total, cytoplasm, and nuclear) and HO-1 in hippocampal neurons exposed to 20 μM OxyHb after the silencing of KLF6 determined by Western blot analysis. **(E)** OxyHb-induced hippocampal neuronal apoptosis after different infection determined by flow cytometry. **(F)** The changes of mitochondrial active oxygen determined by MitoSOX fluorescent staining. **(G)** Changes in mitochondrial membrane potential determined by JC-1 staining. *p < 0.05. The measurement data were expressed as mean ± standard deviation. Comparisons between two groups were analyzed using unpaired *t*-test, and comparisons among multiple groups were tested by one-way ANOVA followed by Dunnett *post-hoc* test. The experiment was repeated three times.

The expression of Nrf2 and HO-1 in the hippocampus of ICH rats was determined by IHC. It was observed that the positive cells of Nrf2 and HO-1 in ICH rats increased (Figure 7A). Furthermore, RT-qPCR demonstrated the expression of SIRT5 increased notably after oe-SIRT5 treatment but was diminished after sh-SIRT5 treatment in neurons (Figure 7B). Then, in order to further explore whether KLF6 regulated the Nrf2/HO-1 signaling pathway by regulating SIRT5 in ICH, we infected the hippocampal neurons with the lentivirus carrying sh-KLF6 and oe-KLF6. Western blot analysis documented decline of the levels of SIRT5 and cytoplasmic Nrf2 and elevation of levels of total Nrf2, HO-1, and nuclear Nrf2 in hippocampal neurons infected with oe-KLF6, which was opposite in hippocampal neurons infected with sh-KLF6 (Figure 7C). These results illustrated that KLF6 promoted activation of the Nrf2/HO-1 signaling pathway by downregulating the SIRT5 expression in hippocampal neurons.

Further, hippocampal neurons were treated by OxyHb. It was documented that OxyHb treatment activated the protein levels of total Nrf2, HO-1, and nuclear Nrf2 and inhibited the levels of SIRT5 and cytoplasmic Nrf2 in the hippocampal neurons, which was nullified by further treatment with sh-KLF6. In the presence of sh-KLF6, sh-SIRT5 contributed to upregulated SIRT5 and cytoplasmic Nrf2 and downregulated total Nrf2, HO-1, and nuclear Nrf2 in OxyHb-induced neurons (Figure 7D).

Flow cytometric analysis revealed that OxyHb treatment promoted cell apoptosis, and oe-SIRT5 treatment diminished OxyHb-caused apoptosis rate of hippocampal neurons. However, the apoptosis rate was increased in hippocampal neurons treated by OxyHb + sh-SIRT5, which was counteracted by sh-KLF6 (Figure 7E). For MitoSOX fluorescence staining, the MitoSOX fluorescence intensity of OxyHb-induced neurons was notably declined by oe-SIRT5, but was remarkably augmented by sh-SIRT5. The increase of MitoSOX fluorescence intensity of OxyHb-induced neurons caused by sh-SIRT5 was normalized by treatment with sh-KLF6 (Figure 7F). JC-1 staining exhibited that the red fluorescence intensity was elevated, the green fluorescence intensity was diminished, and the mitochondrial membrane potential was enhanced in OxyHb-induced neurons by overexpressing SIRT5, which was opposite after silencing SIRT5. The sh-KLF6 treatment normalized the changes of mitochondrial membrane potential of OxyHb-induced neurons caused by sh-SIRT5 (Figure 7G).

Taken together, KLF6 silencing inactivated the Nrf2/HO-1 signaling pathway in OxyHb-induced hippocampal neurons through SIRT5, inhibiting hippocampal neuronal apoptosis, mitochondrial ROS, and mitochondrial damage.

### KLF6 Promoted Brain Tissue Damage in ICH Rats by Inhibiting SIRT5 and Activating the Nrf2/HO-1 Signaling Pathway

**Figure 8:**
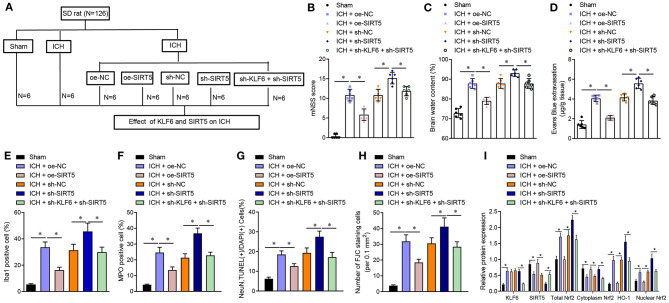
KLF6 accelerates neurological dysfunction and hippocampal neuronal apoptosis and degeneration in ICH rats through SIRT5/Nrf2/HO-1 axis. **(A)** Different infection of ICH model rats. ICH rats were treated with oe-NC, oe-SIRT5, sh-NC, sh-SIRT5, and sh-SIRT5 + sh-KLF6. **(B)** The mNSS score of ICH rats. **(C)** Water content in brain tissues of ICH rats. **(D)** Evans blue penetration of brain tissues of ICH rats. **(E)** Microglia activation of ICH rats detected by immunofluorescence staining. **(F)** The activation of neutrophils of ICH rats detected by immunofluorescence staining. **(G)** The apoptosis of hippocampal neurons of ICH rats detected by TUNEL staining. **(H)** The degeneration of hippocampal neurons in the cerebral cortex of ICH rats detected by FJC staining. **(I)** The levels of KLF6, SIRT5, Nrf2 (total, cytoplasm, and nucleus), and HO-1 in the hippocampus of ICH rats detected by Western blot analysis. *n* = 6 rats/group. *p < 0.05. The measurement data were expressed as mean ± standard deviation. Comparisons between two groups were analyzed using unpaired *t*-test, and comparisons among multiple groups were tested by one-way ANOVA followed by Dunnett *post-hoc* test.

To further verify whether KLF6 activated the Nrf2/HO-1 signaling pathway to promote ICH injury through SIRT5, ICH rats were treated with oe-NC, oe-SIRT5, sh-NC, sh-SIRT5, and sh-SIRT5 + sh-KLF6 (Figure 8A). The mNSS score of ICH rats transduced with oe-SIRT5 was lowered. However, the mNSS score of ICH rats treated with sh-SIRT5 was augmented, which was abrogated by sh-KLF6 treatment (Figure 8B). The cerebral water content of ICH rats transduced with oe-SIRT5 was reduced. On the contrary, the cerebral water content was enhanced in ICH rats infected with sh-SIRT5, which was reversed by further silencing KLF6 (Figure 8C). As for EB staining, the penetration of EB was reduced in ICH rats transduced with oe-SIRT5, but was increased in ICH rats infected with sh-SIRT5. The penetration of EB in ICH rats was diminished by silencing KLF6 in the presence of sh-SIRT5 (Figure 8D). Based on immunofluorescence staining results, the percentage of Iba1^+^ and MPO^+^ cells in ICH rats was lowered after overexpression of SIRT5 but was enhanced after silencing of SIRT5. Silencing KLF6 led to reduction of the percentage of Iba1^+^ and MPO^+^ cells in ICH rats in the presence of sh-SIRT5 (Figures 8E,F).

TUNEL staining and FJC staining displayed that the apoptosis rate and degeneration of hippocampal neurons in ICH rats transduced with oe-SIRT5 were reduced, which was opposite after treatment with sh-SIRT5. In the presence of sh-SIRT5, the apoptosis rate and the degeneration of hippocampal neurons in ICH rats were diminished by infection with sh-KLF6 (Figures 8G,H). Western blot analysis revealed that the expression of KLF6 in ICH rats transduced with oe-SIRT5 remained unchanged, the expression of SIRT5 and cytoplasmic Nrf2 increased, and the total Nrf2, HO-1, and nuclear Nrf2 levels were reduced, which was opposite in ICH rats infected with sh-SIRT5. The expression of KLF6 in ICH rats infected with sh-SIRT5 was lower than that in ICH rats infected with sh-KLF6 + sh-SIRT5, along with higher SIRT5 and cytoplasmic Nrf2 expression and lower total Nrf2, HO-1, and nuclear Nrf2 levels (Figure 8I).

In conclusion, KLF6 activated the Nrf2/HO-1 signaling pathway through SIRT5 to promote neurological dysfunction, neuroinflammation, and hippocampal neuronal apoptosis and degeneration after ICH.

### KLF6 Promoted Oxidative Stress in the Hippocampus of ICH Rats Through the SIRT5/Nrf2/HO-1 Axis

**Figure 9:**
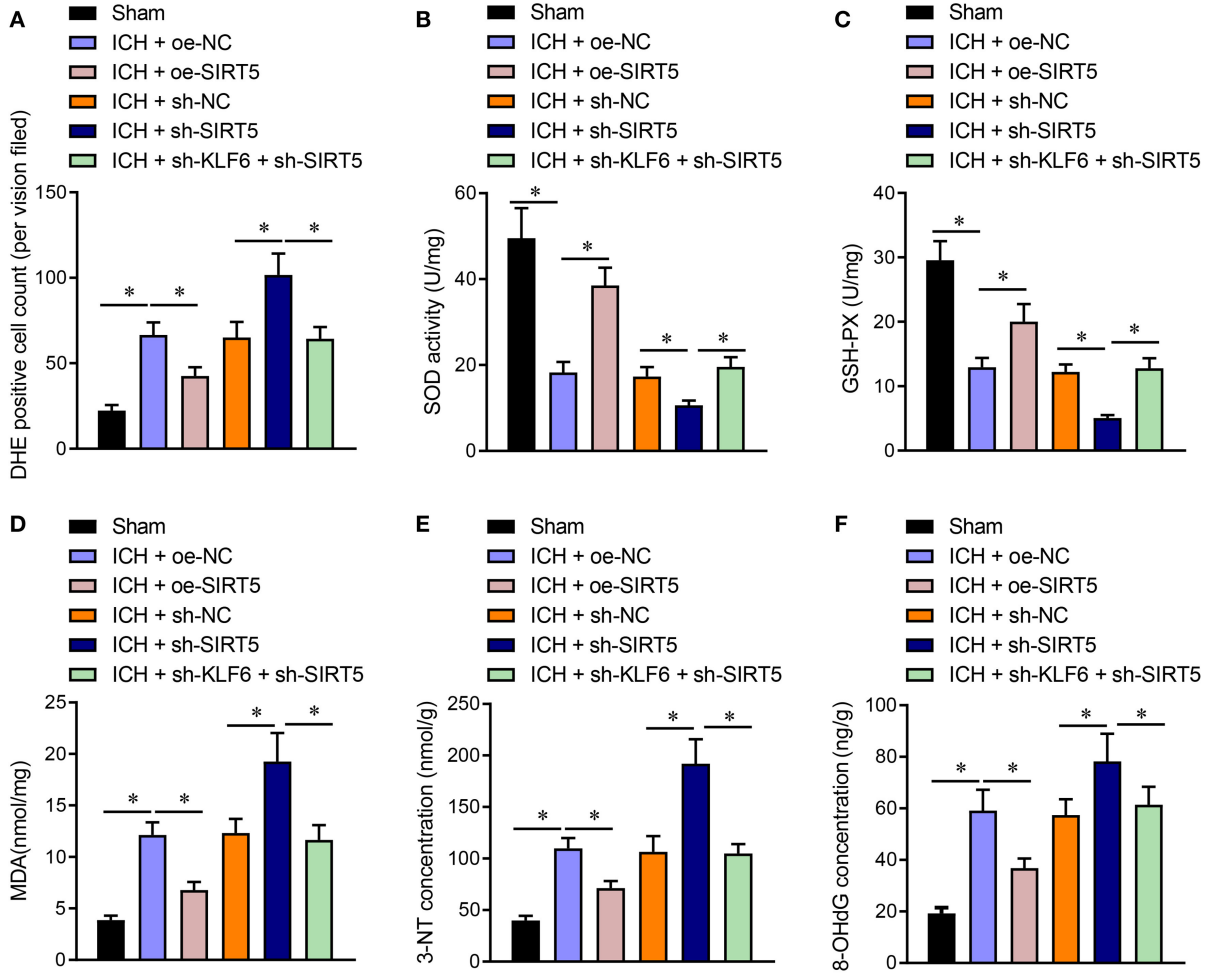
KLF6 silencing depresses oxidative stress in ICH rats by inhibiting SIRT5-mediated Nrf2/HO-1 signaling pathway. ICH rats were infected lentiviruses carrying oe-NC, oe-SIRT5, sh-NC, sh-SIRT5, and sh-SIRT5 + sh-KLF6. **(A)** The active oxygen in the hippocampus of ICH rats determined by DHE staining. **(B)** SOD activity in hippocampal tissues of ICH rats. **(C)** GSH-Px activity in hippocampal tissues of ICH rats. **(D)** MDA level in hippocampal tissues of ICH rats. **(E)** 3-NT concentration in hippocampus of ICH rats determined by ELISA. **(F)** 8-OHdG concentration in hippocampus of ICH rats determined by ELISA. *n* = 6 rats/group, *p < 0.05. The measurement data were expressed as mean ± standard deviation. Comparisons between two groups were analyzed using unpaired *t*-test, and comparisons among multiple groups were tested by one-way ANOVA followed by Dunnett *post-hoc* test.

The oxidative stress levels in the hippocampus of rats were detected by DHE staining. The results showed that the number of DHE-positive cells in ICH rats transduced with oe-SIRT5 was diminished. The number of DHE-positive cells was increased in ICH rats infected with sh-SIRT5, which was annulled by sh-KLF6 (Figure 9A). Furthermore, SOD and GSH-Px enzyme activity was enhanced, whereas the MDA content was lowered in ICH rats overexpressing SIRT5, which was opposite in ICH rats after silencing SIRT5. Compared with SIRT5 silenced ICH rats, the SOD and GSH-Px enzyme activities were augmented, whereas the MDA content was reduced in ICH rats treated with sh-KLF6 + sh-SIRT5 (Figures 9B–D). ELISA data documented that 3-NT and 8-OHdG contents were diminished in the hippocampus of SIRT5-overexpressed ICH rats. The 3-NT and 8-OHdG contents were promoted in SIRT5-silenced ICH rats, which was nullified by silencing KLF6 (Figures 9E,F). Hence, KLF6 activated the Nrf2/HO-1 signaling pathway through SIRT5 to promote oxidative stress in rats after ICH.

## Discussion

ICH is a leading cause of stroke-related mortality and morbidity that lacks effective acute or preventive treatments (Hostettler et al., 2019). Previous studies identified that edema formation, neuronal apoptosis, oxidative stress, and increased blood–brain barrier permeability were key factors contributing to ICH-induced brain damage and the poor outcome of ICH (Iniaghe et al., 2015; Wei et al., 2017). It has been illustrated that KLF6 was an essential mediator for central nerve system myelination (Laitman et al., 2016). Hence, the role of KLF6 in ICH and the involved downstream mechanisms were investigated in the current study. The experimental data elucidated the promoting effect of KLF6 on oxidative stress and neurological dysfunction after ICH through the activation of the Nrf2/HO-1 signaling pathway by downregulating SIRT5.

Primarily, it was observed in this study that KLF6 was highly expressed in hippocampal tissues of rats after ICH modeling. Besides, KLF6 downregulation resulted in enhancement of SOD and GSH-Px enzyme activity and decline of MDA content and 3-NT and 8-OHdG expression, indicating that oxidative stress was relieved, which further alleviated ICH. Consistent with our finding, other researchers also revealed that the aberrantly high expression of KLF6 in the mouse hippocampus was responsible for the glial responses after status epilepticus (Jeong et al., 2011). Besides, upregulation of KLF6 was also observed in injured hypoglossal motor neurons after neurological dysfunction, and it was correlated with nerve regeneration (Nagata et al., 2014). Furthermore, another study confirmed that KLF6 exerted a promoting effect in macrophage inflammatory and hypoxic response (Kim et al., 2020). SOD, MDA, 3-NT, 8-OHdG, and GSH-Px are renowned oxidative stress indicators (Zhao et al., 2019; Bandookwala and Sengupta, 2020; Lv et al., 2020). Moreover, a prior study confirmed that the increased expression of SOD and GSH-Px and the diminished expression of MDA, 3-NT and 8-OHdG represented the alleviated oxidative stress (Liu et al., 2019, 2020). Taken together, these findings supported the protective effect of KLF6 silencing against oxidative stress after ICH.

The present study further illustrated that SIRT5 was poorly expressed in hippocampal tissues of rats after ICH modeling and KLF6 activated the Nrf2/HO-1 signaling pathway through SIRT5 downregulation, thereby exacerbated oxidative stress and neuronal apoptosis after ICH. SIRT5 is an essential regulator of mitochondria that contributes to neurological diseases, and SIRT5 silencing remarkably worsened hippocampal neuronal loss and degeneration (Li and Liu, 2016). Similarly, an obvious downregulation of SIRT6 was observed in hippocampus and saliva of patients with Alzheimer disease, which is also a disease in close relation to oxidative stress (Pukhalskaia et al., 2020). Meanwhile, another study confirmed the binding relationship between KLF6 and SIRT5, and SIRT5 was reported to be repressed by KLF6 through transcription (Hong et al., 2019). In line with our findings, it was also investigated that Nrf2/HO-1 inactivation contributed to the attenuated oxidative stress in kidney injury (Mahran, 2020). More importantly, Nrf2/HO-1 signaling pathway was demonstrated to be regulated by SIRT5 and thus regulated cisplatin-induced DNA damage in a ROS-dependent manner (Sun et al., 2019). On a nutshell, it is safe to say the activation of Nrf2/HO-1 signaling pathway triggered by KLF6-mediated SIRT5 repression promoted the neurological dysfunction and oxidative stress in rats after ICH.

## Conclusion

**Figure 10:**
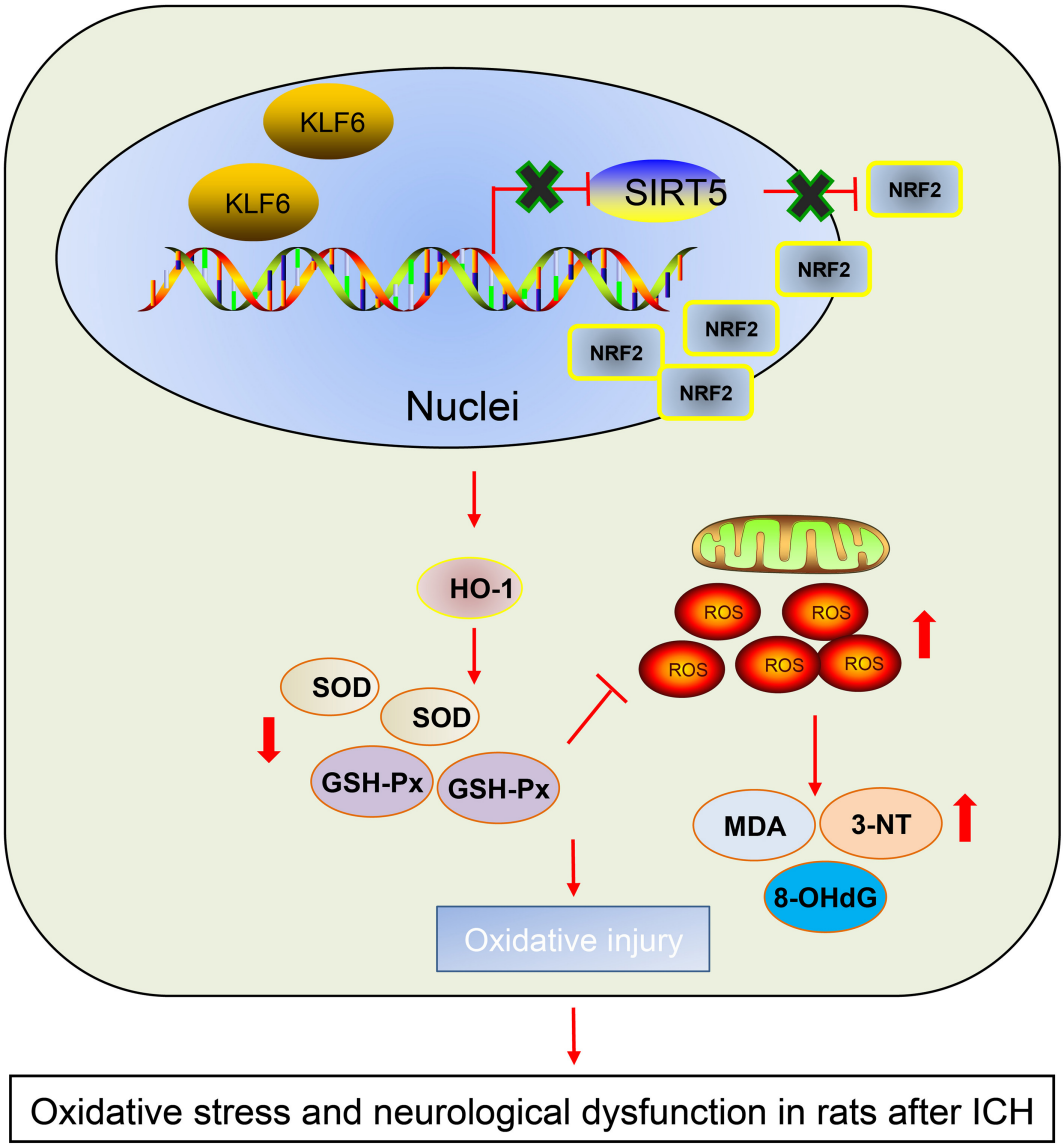
The graphical summary of the function and mechanism of KLF6 in ICH. Nuclear transcription factor KLF6 mediates SIRT5/Nrf2/HO-1 axis to regulate brain tissue damage and oxidative stress in ICH rats. Nuclear transcription factor KLF6 is highly expressed in ICH rats. KLF6 transcriptionally inhibits SIRT5 and then activates the Nrf2/HO-1 signaling pathway, thus repressing mitochondrial membrane potential and promoting hippocampal ROS and apoptosis in neurons, which ultimately accelerates oxidative stress and neurological dysfunction in rats after ICH.

According to aforementioned findings, it was demonstrated that KLF6 silencing alleviated oxidative stress and neurological dysfunction in ICH by suppressing of Nrf2/HO-1 signaling pathway through upregulation of SIRT5 (Figure 10). This study proposed the implication of KLF6-mediated SIRT5/Nrf2/HO-1 axis in oxidative stress and neurological dysfunction after ICH, which represented a promising therapeutic method for ICH. However, other available molecular mechanisms are expected to be identified for better clinical outcome of ICH management.

## Data Availability Statement

The datasets presented in this study can be found in online repositories. The names of the repository/repositories and accession number(s) can be found in the article/Supplementary Material.

## Ethics Statement

The animal study was reviewed and approved by the Animal Care and Use Committee of Shenzhen Beike Biotechnology Research Institute.

## Author Contributions

JS conceived and designed the research. JCa performed the experiments. JCh interpreted the results of experiments. SL analyzed the data. XL prepared the figures. YH drafted the paper. XC and SH edited and revised the manuscript. All authors read and approved the final version of manuscript.

## Conflict of Interest

The authors declare that the research was conducted in the absence of any commercial or financial relationships that could be construed as a potential conflict of interest.
