# Supplementary material for: Krüppel-Like Factor 6 Silencing Prevents Oxidative Stress and Neurological Dysfunction Following Intracerebral Hemorrhage via Sirtuin 5/Nrf2/HO-1 Axis
Source: Front Aging Neurosci. 2021 Jun 3;13:646729. doi: 10.3389/fnagi.2021.646729 (PMC8209425; doi:10.3389/fnagi.2021.646729)
Supplement: Supplementary file 1 [file Table_1.docx]

**Supplementary table 1** Primer sequences of RT-qPCR

| Gene | Species | Primer sequence |
| --- | --- | --- |
| KLF6 | Rno | F: 5’-ACGACCAAGTTTACCTCTGAC-3’ |
|  |  | R: 5’-CAGCCCCATAGTTGAGAAGAT-3’ |
| SIRT5 |  | F: 5’-TGTCTGGGACCTGGTGCGGT-3’ |
|  |  | R: 5’-AAGGCTTCTCTCCGGTGTGCGT-3’ |
| GAPDH |  | F: 5’-GACATCAAGAAGGTGGTGAA-3’ |
|  |  | R: 5’-TGTCATACCAGGAAATGAGC-3’ |

Note: KLF6, kruppel like factor 6; SIRT5, sirtuin 5; GAPDH, glyceraldehyde-3-phosphate dehydrogenase; F, forward; R, reversed; RT-qPCR, reverse transcription-quantitative polymerase chain reaction
