# Supplementary material for: Krüppel-Like Factor 6 Silencing Prevents Oxidative Stress and Neurological Dysfunction Following Intracerebral Hemorrhage via Sirtuin 5/Nrf2/HO-1 Axis
Source: Front Aging Neurosci. 2021 Jun 3;13:646729. doi: 10.3389/fnagi.2021.646729 (PMC8209425; doi:10.3389/fnagi.2021.646729)
Supplement: Supplementary file 2 [file Table_2.docx]

**Supplementary table 2** Primer sequences for ChIP assay

| Primers | Sequence |
| --- | --- |
| Primer 1 | F: 5’-ACACCGGGAAACGTTCTTTG-3’ |
|  | R: 5’-TGAGGCAAGATCCATGGAAGG-3’ |
| Primer 2 | F: 5’-ACGTTCTTTGCCGTAACTCCA-3’ |
|  | R: 5’-AGGCAAGATCCATGGAAGGAC-3’ |

Note: F, forward; R, reversed; ChIP, Chromatin immunoprecipitation
